# Supplementary material for: A cluster-randomized trial of household vs incentive-based tuberculosis contact investigation in rural South Africa: implementation reach
Source: PLOS Glob Public Health. 2025 Dec 5;5(12):e0005580. doi: 10.1371/journal.pgph.0005580 (PMC12680170; doi:10.1371/journal.pgph.0005580)
Supplement: S1 File — S1 Table. Enrollment of index participants (people diagnosed with TB) in each arm. S2 Table. Index participant characteristics associated with enrolling at least one contact person. (DOCX) [file pgph.0005580.s001.docx]

**Supplementary Table 1**. Enrollment of index participants (people diagnosed with TB) in each arm.

|  | **Household-based arm** | **Incentive-based arm** | **Total** |
| --- | --- | --- | --- |
|  | **(N=1269)** | **(N=1294)** | **(N=2563)** |
| **Total participants contacted** | 1217 (95.9) | 1245 (96.2) | 2462 (96.1) |
| Enrolled | 782 (61.6) | 780 (60.3) | 1562 (60.9) |
| Declined | 116 (9.1) | 74 (5.7) | 190 (7.4) |
| Attempts exhausted | 189 (14.9) | 250 (19.3) | 439 (17.1) |
| Mental impairment | 7 (0.6) | 14 (1.1) | 21 (0.8) |
| No study languages spoken | 10 (0.8) | 9 (0.7) | 19 (0.7) |
| Not able to give consent ***^†^*** | 113 (8.9) | 118 (9.1) | 231 (9.0) |
|  |  |  |  |
| **Participants not contacted or missing information** | 52 (4.1) | 49 (3.8) | 101 (3.9) |

***^†^*** Not able to give consent includes the following reasons: death, critical illness, unavailability, or relocation.

**Supplementary Table 2.** Index participant characteristics associated with enrolling at least one contact person.

|  | **Household-based Arm** | | | | | **Incentive-based Arm** | | | | | |
| --- | --- | --- | --- | --- | --- | --- | --- | --- | --- | --- | --- |
|  | **Crude mixed-effects logistic regression** | | **Adjusted mixed-effects logistic regression** | | | **Crude mixed-effects logistic regression** | | **Adjusted mixed-effects logistic regression** | | |  |
|  | **OR *** | **CI *** | | **aOR *** | **CI** | **OR** | **CI** | | **aOR** | **CI** | |
| Female | Ref |  | | Ref |  | Ref |  | | Ref |  | |
| Male | 0.59 | 0.43 – 0.81 | | 0.60 | 0.43 – 0.83 | 0.65 | 0.48 – 0.89 | | 0.65 | 0.48 – 0.89 | |
|  |  |  | |  |  |  |  | |  |  | |
| Age < 18 years | Ref |  | | Ref |  | Ref |  | | Ref |  | |
| Age ≥ 18 years | 0.51 | 0.23 – 1.13 | | 0.58 | 0.26 – 1.27 | 0.31 | 0.17 – 0.57 | | 0.34 | 0.18 – 0.62 | |
|  |  |  | |  |  |  |  | |  |  | |
| Vhembe district | Ref |  | | Ref |  | Ref |  | | Ref |  | |
| Waterberg district | 0.34 | 0.24 – 0.48 | | 0.34 | 0.23 – 0.48 | 0.71 | 0.39 – 1.27 | | 0.75 | 0.41 – 1.34 | |
|  |  |  | |  |  |  |  | |  |  | |
| HIV negative | Ref |  | | Ref |  | Ref |  | | Ref |  | |
| HIV positive | 1.04 | 0.75 – 1.45 | | 1.02 | 0.73 – 1.43 | 0.78 | 0.57 – 1.08 | | 0.80 | 0.57 – 1.12 | |
| HIV status unknown | 1.15 | 0.54 – 2.45 | | 1.08 | 0.50 – 2.32 | 1.21 | 0.58 – 2.53 | | 0.89 | 0.41 – 1.97 | |

Mixed-effects logistic regression models with a random intercept for clinic were adjusted for arm, sex, age, district, tuberculosis symptoms, smoking and HIV status.

OR=odds ratio; aOR=adjusted odds ratio; CI=confidence interval.
