# Supplementary material for: A cluster-randomized trial of household vs incentive-based tuberculosis contact investigation in rural South Africa: implementation reach
Source: PLOS Glob Public Health. 2025 Dec 5;5(12):e0005580. doi: 10.1371/journal.pgph.0005580 (PMC12680170; doi:10.1371/journal.pgph.0005580)
Supplement: Attachment — Submitted filename: Response letter.docx [file pgph.0005580.s002.docx]

Dear Editor-

We thank the reviewers for their time in reading and commenting on our manuscript. In addressing their comments, we feel the manuscript is appreciably strengthened. Below please find our point-by-point response to each reviewer’s comments.

**Reviewer #1: Thank you for this interesting and relevant study, as contact tracing of people with TB represents a large barrier to achieving testing and treatment goals, and the use of incentives in TB services is not well understood.

While the manuscript is well-written and the analysis appears to be sound, my major issue is to do with incomplete sentences, specifically Methods, line 94 and Conclusion: page 22, line 385 ends with "These findings". As a result of not knowing what the missing information entails, I would request the authors resubmit and consider proof-reading before doing so.**

We thank the reviewer for the comment. We apologize for the oversight. We have corrected the noted sections, and carefully read over the manuscript to correct any other such errors.

**Minor comments:
The secondary analysis is restricted to data between July 2016 to January 2018, representing 18 months, but the intervention describes 18 months per intervention in all clinics including a 6 month period of no intervention. This would imply 42 months in total. Did the analysis use the first or second 18 month intervention? Please clarify which period is being analysed or if this has been misunderstood.**

Thank you for the comment. We conducted the study from July 18, 2016 to January 17, 2020. Fourteen clinics were allocated to conduct household-based investigation followed by incentive-based investigation, and the other 14 were allocated to the opposite order. The first period was 18 July 2016 through 17 January 2018, and the second period was 18 July 2018 through 17 January 2020. These two 18-month periods were separated by a 6-month washout period during which no contact investigation activities were performed (between 18 January 2018 and 17 July 2018). We are using data from both 18-month intervention periods. We corrected the Methods section to reflect this:

“This is a secondary analysis of a cluster-randomized trial (Kharitode TB) of TB case finding strategies in Limpopo Province, South Africa from July 18, 2016 to January 17, 2020.”

“Contact investigation was carried out in 28 of the 56 study clinics. Among these, 14 clinics initially implemented the household-based strategy and 14 implemented the incentive-based strategy. Clinics were randomized to one strategy for 18 months and switched to the opposite strategy for 18 months after a 6-month “washout” period during which neither strategy was employed. The present analysis includes data from both 18-month intervention periods.” **The authors describe making home visits when unable to reach participants by phone as a method of overcoming this barrier, however limitations related to relying on contact by mobile phones and possible reasons for this are discussed (line 305-307). Were these reasons documented when study staff were unable to reach participants by phone? Were any other methods for reaching participants considered such as making use of TB tracer teams or community health workers? As these are important aspects of retention in care.**

Thank you for the question. We agree that community health workers play an important role in contact tracing. However, our study did not utilize any external services, as the primary aim was to compare the effectiveness of different contact tracing strategies. All participant follow-up was conducted solely by research staff as the availability of tracer teams or community health workers was variable by district and even by clinic.

Reasons for being unreachable were documented and are presented in Supplementary Table 1.

**Concerning lower participation among people living with HIV, is it known whether or not these people were already engaged in chronic ART care and may have already had contacts screened for TB?**

We do not have information about ART among participants with HIV. We added this in the Limitations sub-section:

“Antiretroviral therapy (ART) status of index participants and contact persons with HIV was not known and it was not possible to understand if ART engagement affected study participation differentially.”

**Reviewer #2: This study is an embedded study from a cluster randomized trial. The cluster randomized trial randomized 56 study clinics to facility based screening or contact investigation in South Africa. Here, they consider the 28 clinics randomized to contact investigation. They compare 14 with traditional contact investigation to 14 with incentive based contact investigation on the TB cascade of diagnosis. The comparison lasted for 18 months, then after a 6 month washout period, facilities switched interventions. They compared the proportion of individuals completing each step of the contact investigation cascade.**

**All statistical tests should take into account the clustering by facility, and possibly clustering by index patient. Observations are not independent.**

We agree with the reviewer and implemented mixed models in our primary analysis to account for clustering by facility. Clustering by index patient was not possible as many index patients had only one contact person.

From the methods: “We included study arm as a fixed effect in the mixed-effects logistic regression models to directly assess differences between arms while accounting for clustering at the clinic level.”

Please see Table 4 in the revised manuscript for the updated model results accounting for clustering by facility. We would like to note that there were no significant differences from the previously presented models where clustering was not accounted for.

**It is not clear how the crossover nature of the data was dealt with. Please clarify.**

Thank you for the question. Because this secondary analysis was descriptive and focused on implementation reach rather than comparative effectiveness, we decided to combine the data from both periods without adjustment for period effects. The clarification was added to the Data Analysis sub-section:

“Data from both 18-month intervention periods were combined for analysis, as this secondary analysis was descriptive and aimed to evaluate overall implementation reach rather than intervention effects.”

**1. Please justify using the pooled number of individuals for the denominator of the proportion of index participants enrolled within 2 months of TB dx.**

We pooled the sample of the two arms for index participants because enrollment procedures for index participants were identical across both strategies.

**2. The proportion of index participants for whom at least one contact person was screened was compared between arms via chi-squared test - this is not appropriate. Clustering must be accounted for. Similarly, for all the other proportions.**

We thank the reviewer for this comment. In response, we have removed the unadjusted chi-squared tests comparing proportions between study arms. The proportions presented in the manuscript are now reported purely as descriptive summaries to illustrate overall implementation reach. Formal comparisons between arms, which account for clustering at the clinic level and adjust for covariates, are presented in the mixed-effects logistic regression models described in the Methods (see Table 4 for the mixed effects logistic model results).

**3. There may also be clustering by index patient for the other proportions based on contacts. This clustering should be accounted for.**

We considered clustering at the index level. However, many index participants had only one enrolled contact, making estimation of clustering by index participant unstable or impossible. Therefore, our primary models (see Table 4) accounted only for clustering at the clinic level.

**4. For the multivariable models used to explore index participant characteristics -- mixed logistic models should be used to account for clustering by clinic.**

Thank you for the suggestion. We redid our analyses using mixed models and now our results account for clustering by clinic (see Table 4).

**5. For the multivariable models used to explore contact participant characteristics -- models that allow for clustering by clinic and by index patient should be used.**

Thank you for the suggestion. We redid all our analyses using mixed models and now our results account for clustering by clinic. However, clustering by index participant was not feasible as many index participants had only one contact person.

**6. In all tables (including supplemental ones), just present a CI. No need to also present a p value.**

Thank you for the comment. We removed p values from the tables and present confidence intervals only. **7. In the discussion, it is emphasized that fewer than half of potential index patients had at least one contact screened and that this was lower in the incentive arm than the household based arm. However, this seems to ignore the fact that the incentive based arm did a lot better at continuing to the end - overall 0.75=953/1269 contacts notified of results per potential index cases in the household arm vs. 1.07=1391/1294 in the incentive arm. This seems pretty important yet is not discussed - why?**

Thank you for highlighting this important piece. The enrollment of contact persons was lower in the incentive-based arm. However, among those who enrolled, study reach was higher. We highlighted this in the Discussion section, as this is indeed an important finding of our analysis:

“Among enrolled contact persons, implementation reach was higher in the incentive-based arm, with over two-thirds of all contact persons enrolled notified of their Xpert MTB/RIF results as opposed to only half in household-based arm.” **8. The Conclusions section is cutoff at the end.**

Thank you for the comment. We edited the Conclusion section.

**9. Please specify which variables were included in the models in a footnote in Suppl Table 2.**

Thank you for the comment. We added a footnote specifying variables included in the models for all tables. **10. Table 3 seems a bit repetitive with Table 4. Remove the p-values from Table 3 - these comparisons are presented in Table 4.**

Thank you for the advice, we removed p values from the Table 3. **11. For Table 4 - is it interesting to compare the characteristics by arm?**

Thank you for the suggestion. We redid the analysis to be able to compare sputum collection by arm. We clustered the analysis by clinic, and checked the interaction between arm and HIV, which showed no differenced by arm.

From the Results: “We included study arm as a fixed effect in the mixed-effects logistic regression models to directly assess differences between arms while accounting for clustering at the clinic level.”

**12. Everywhere - instead of univariate and multivariable use "Crude" and "Adjusted".**

Thank you for the comment, we edited the text accordingly.

**Reviewer #3: This well written manuscript contains important information that could guide the Household Contact TB programs in SA. Here are minor comments for consideration.

1. Were all participants reimbursed? Was the reimbursement separate from the incentive?**

Thank you for the question. We compensated all participants for their time for research purposes. Additionally, participants in the incentive-based arm received financial incentives for referring contacts who presented for screening as described in the Methods section, however those in the household arm received no financial incentives for contact investigation.

**2. What was the incentive? This is not mentioned in the manuscript. It may have been described elsewhere but it is also worthwhile to briefly describe it in this manuscript and then provide a citation.**

We provided financial incentives to index participants and their contact persons in the incentive-based arm: each contact person presenting to the clinic for TB screening within two months of index participant enrollment was given 50 South African Rand (ZAR), approximately equal to 3.50 USD. Index participants were reimbursed 20 ZAR, (1.50 USD) for each contact person presenting for screening, and 100 ZAR (7.00 USD) for every contact person diagnosed with TB. This is explained in the Methods section.

**3.Line 152 indicates that some diagnostic outcomes were assessed. However, i have not seen any diagnostic outcomes reported in the results.**

Thank you for this comment. The reviewer is right, we do not assess diagnostic outcomes in this manuscript. We have edited the Methods section accordingly.

**4. Were the people living with HIV on ART? Sometimes, PLHW on ART are unable to produce sputum due to their generally improved health. Table 4 indicates a strong association between being HIV positive and sputum collection/production and i was just wondering how this association would have looked like with PLWH on established ART vs newly on ART.**

As stated above, we do not have information about ART among participants living with HIV. We added this in the Limitations sub-section:

“Antiretroviral therapy (ART) status of index participants and contact persons living with HIV was not known and it was not possible to understand if ART engagement affected study participation differentially.”

**5. Table 4 also shows a strong association with cough and sputum collection and no association with other TB symptoms. What would be the implications of subclinical TB on household contact programs if there were no TB symptoms?**

The strong association between cough and successful sputum collection reflects the practical challenge of obtaining sputum from asymptomatic individuals. As a result, household contact programs that rely on sputum collection may under-detect subclinical TB.

**6. Table 1: What are the "other" TB symptoms? elaborate in a footnote**

Thank you for the clarification request. We have added the information in a footnote.

**7. Were the HIV status and smoking status self-reported? if yes, what are the limitations for these?**

HIV status for index participants was obtained from clinical records. HIV and smoking status for contact persons were self-reported. As there are high levels of stigma associated with HIV in South Africa, this may have resulted in under-reporting HIV status. However, we do not expect this to differ systematically between study arms.

**8. Include a citation for R software**

We included a citation for R software.

**9. Include a reference number for Wits HREC ethics approval.**

Thank you for the suggestion. We included University of the Witwatersrand in South Africa HREC ethics approval number.
